# Supplementary material for: Cohesin Components Stag1 and Stag2 Differentially Influence Haematopoietic Mesoderm Development in Zebrafish Embryos
Source: Front Cell Dev Biol. 2020 Dec 7;8:617545. doi: 10.3389/fcell.2020.617545 (PMC7750468; doi:10.3389/fcell.2020.617545)
Supplement: Supplementary file 1 [file Data_Sheet_1.PDF]

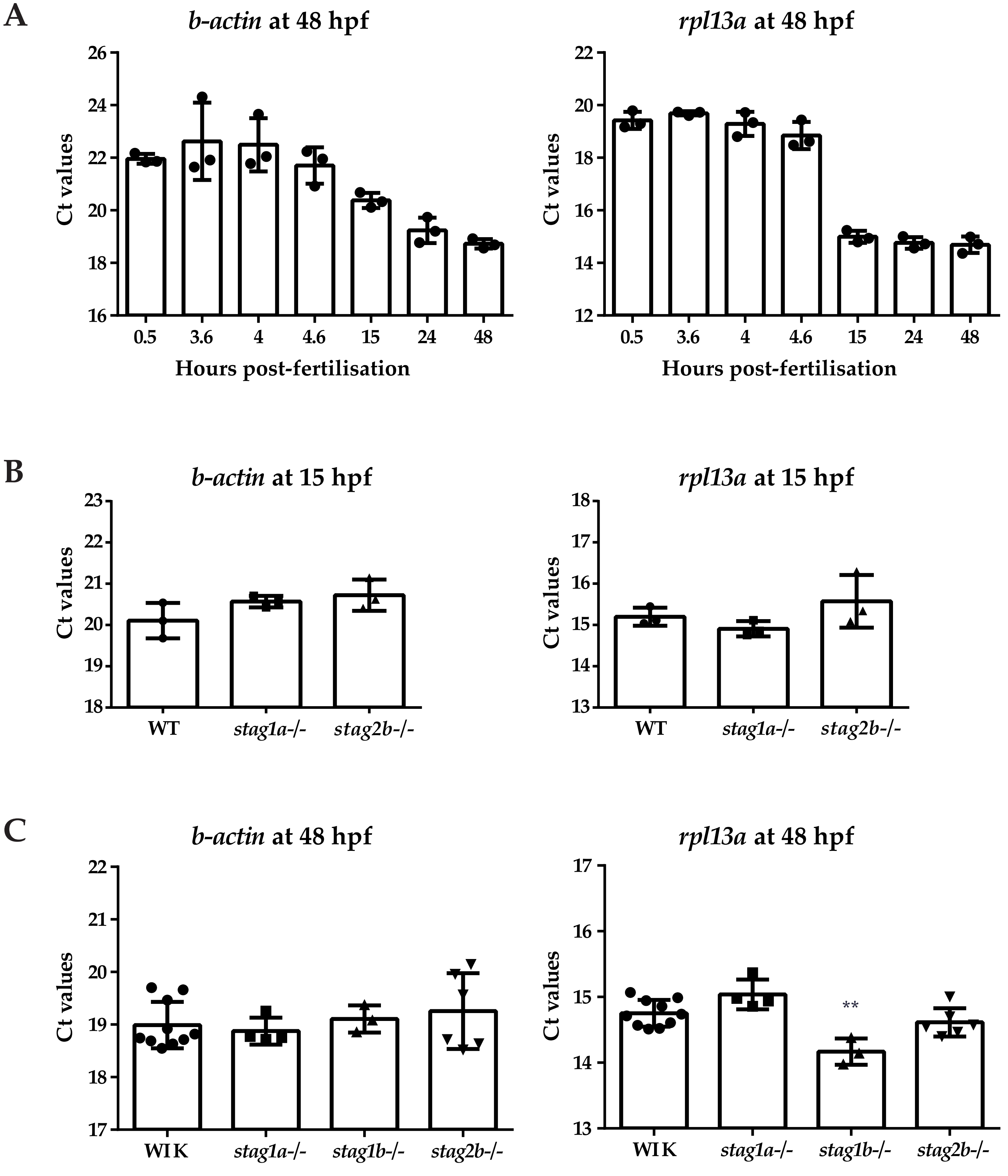

Supplementary Figure 1. Stability of reference genes used for quantitative RT-PCR (qPCR) normalisation. (A) Ct values of *b-actin* and *rpl13a* in wildtype embryos at 48 hpf. **(B)** Ct values of *b-actin* and *rpl13a* in wildtype and mutant embryos at 15 hpf. **(C)** Ct values of *b-actin* and *rpl13a* in wildtype and mutant embryos at 48 hpf. Expression of *rpl13a* is altered in mutants. $^{**}P \leq 0.01$; one-way ANOVA.
