# Supplementary material for: Cohesin Components Stag1 and Stag2 Differentially Influence Haematopoietic Mesoderm Development in Zebrafish Embryos
Source: Front Cell Dev Biol. 2020 Dec 7;8:617545. doi: 10.3389/fcell.2020.617545 (PMC7750468; doi:10.3389/fcell.2020.617545)
Supplement: Supplementary file 2 [file Data_Sheet_2.PDF]

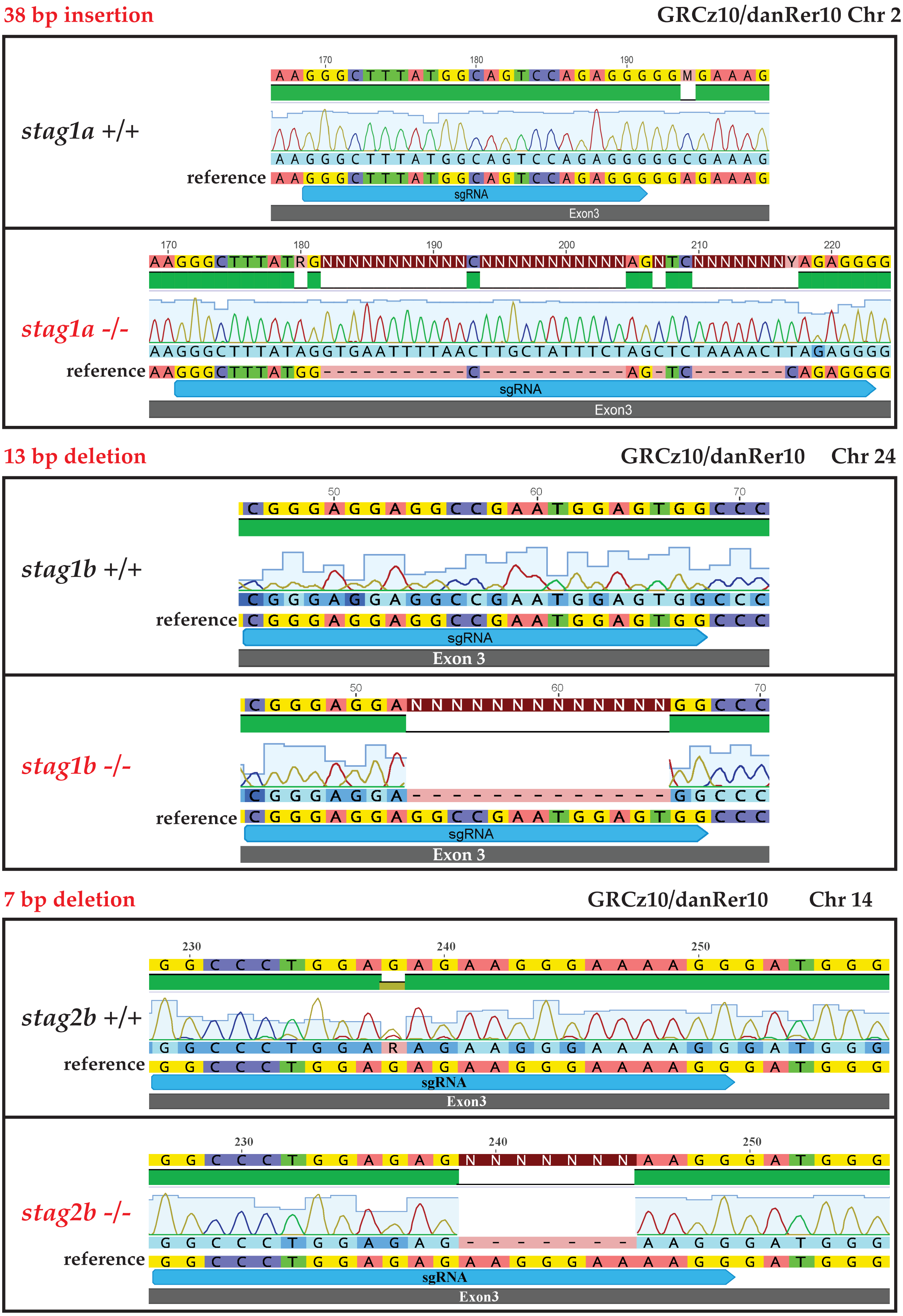

Supplementary Figure 2. Genomic DNA sequence detail of zebrafish *stag* gene germline CRISPR mutants. Nucleotide alignments of wildtype and mutant homozygous sequences are shown. The sgRNA sites are annotated in blue.
