# Supplementary material for: Cohesin Components Stag1 and Stag2 Differentially Influence Haematopoietic Mesoderm Development in Zebrafish Embryos
Source: Front Cell Dev Biol. 2020 Dec 7;8:617545. doi: 10.3389/fcell.2020.617545 (PMC7750468; doi:10.3389/fcell.2020.617545)
Supplement: Supplementary file 3 [file Data_Sheet_3.PDF]

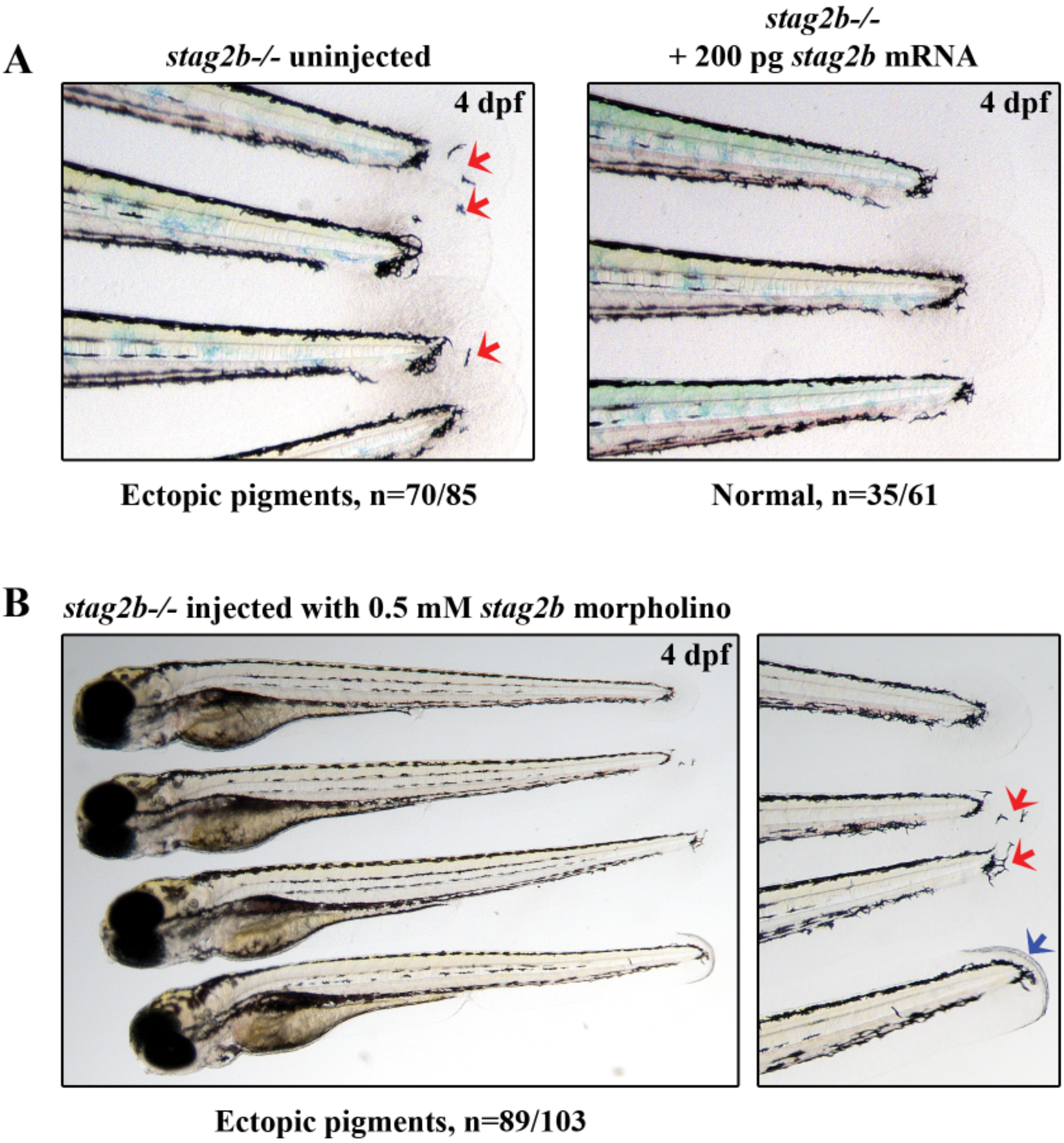

Supplementary Figure 3. The *stag2b* mutation: confirmation of loss of function and and mutant phenotype. (A) The ectopic pigment cells seen in the tail fin of *stag2b*-/- embryos (red arrows) are rescued upon injection of *stag2b* mRNA. Lateral views of tail fin zoom-ins are shown, anterior to the left. **(B)** Injection of 0.5 mM *stag2b* morpholino does not induce any additional phenotypes in *stag2b*-/- embryos, and confirms that Stag2 loss causes displaced pigment cells (red arrows) and tail fin folds (blue arrow). Lateral views of full-length embryos and tail fin zoom-ins are shown, anterior to the left. Numbers of embryos are indicated below the respective panels.
