# Supplementary material for: Cohesin Components Stag1 and Stag2 Differentially Influence Haematopoietic Mesoderm Development in Zebrafish Embryos
Source: Front Cell Dev Biol. 2020 Dec 7;8:617545. doi: 10.3389/fcell.2020.617545 (PMC7750468; doi:10.3389/fcell.2020.617545)
Supplement: Supplementary file 4 [file Data_Sheet_4.PDF]

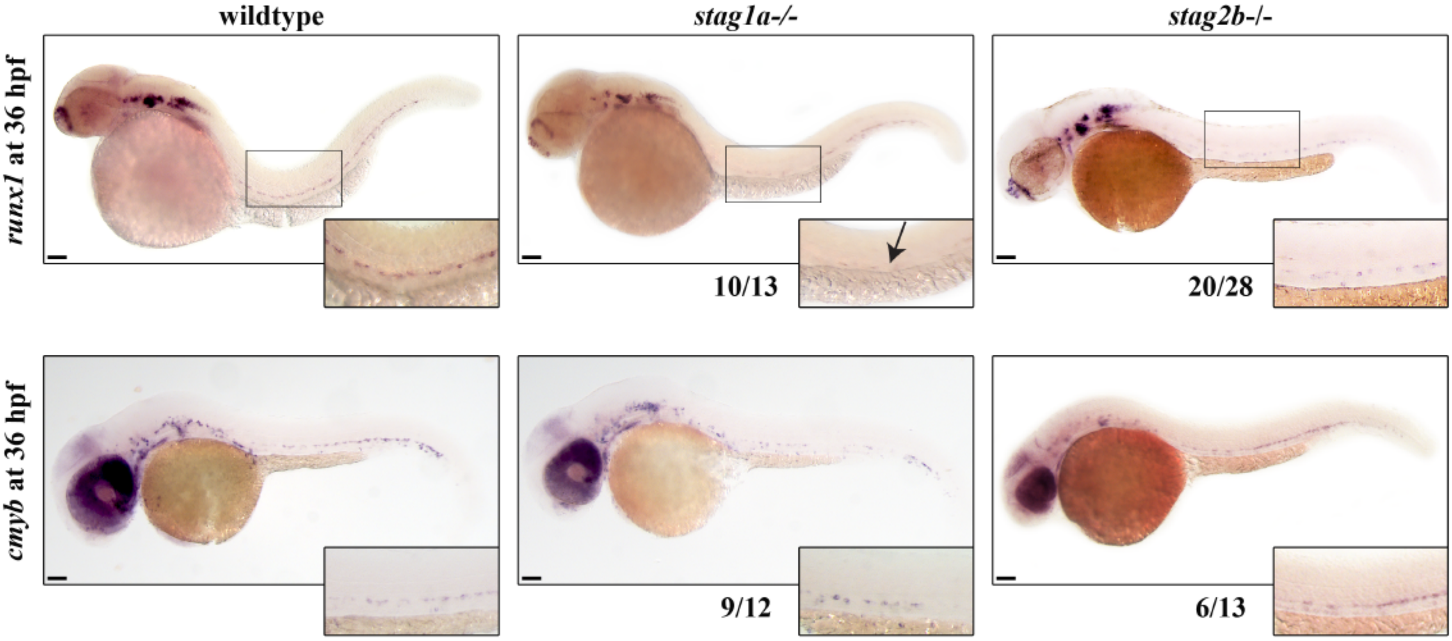

Supplementary Figure 4. Whole-mount *in situ* hybridization analysis of expression of *runx1* and *cmyb* at 36 hpf. Lateral views are shown, anterior to the left. Insets show zoom-ins of the dorsal aorta region. Reduced *runx1* expression in *stag1a*-/- embryos is indicated by an arrow. Number of embryos is indicated below the respective panels. Scale bars are 100 μm.
