# Supplementary material for: Cohesin Components Stag1 and Stag2 Differentially Influence Haematopoietic Mesoderm Development in Zebrafish Embryos
Source: Front Cell Dev Biol. 2020 Dec 7;8:617545. doi: 10.3389/fcell.2020.617545 (PMC7750468; doi:10.3389/fcell.2020.617545)
Supplement: Supplementary file 5 [file Data_Sheet_5.PDF]

A

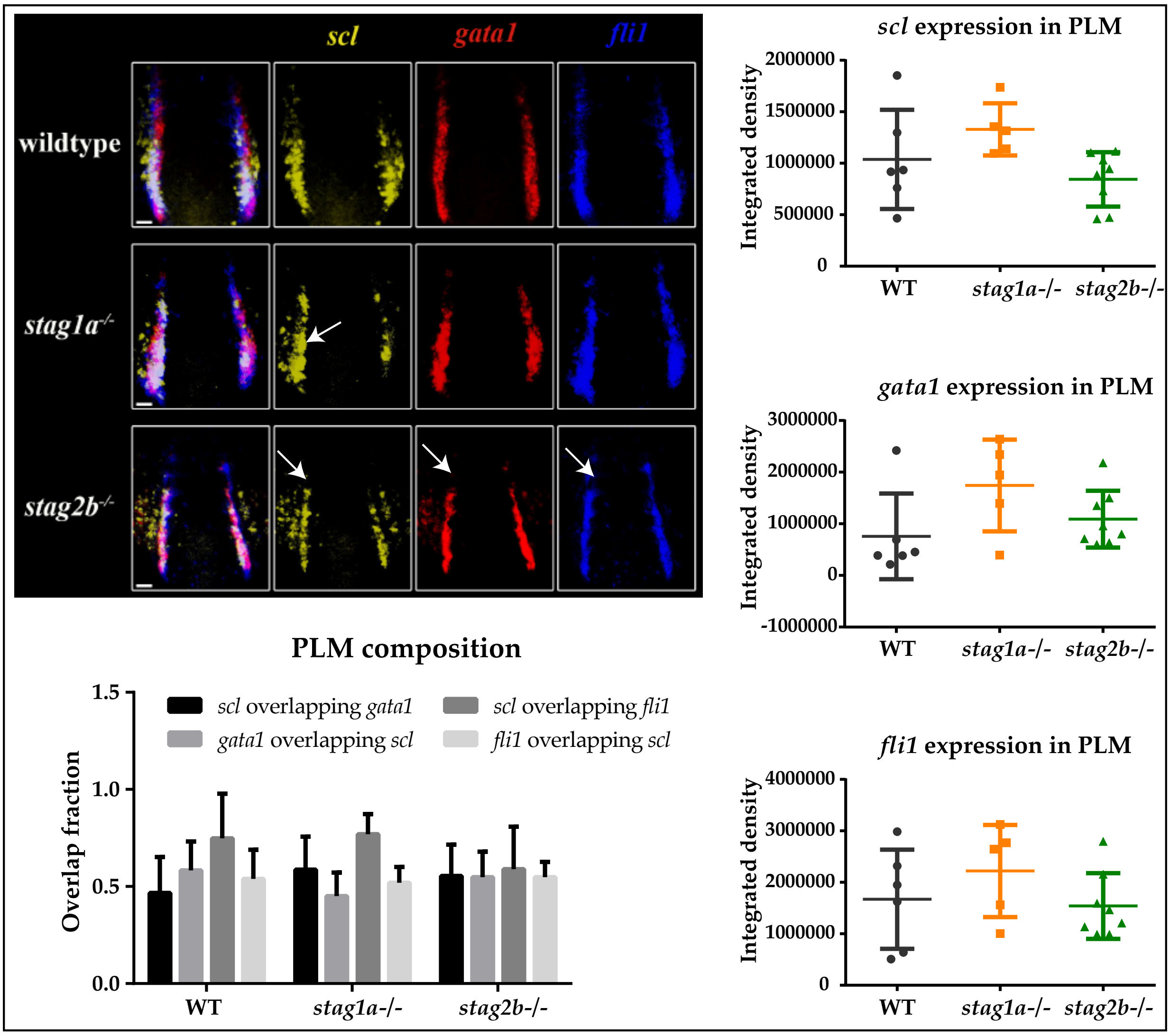

B

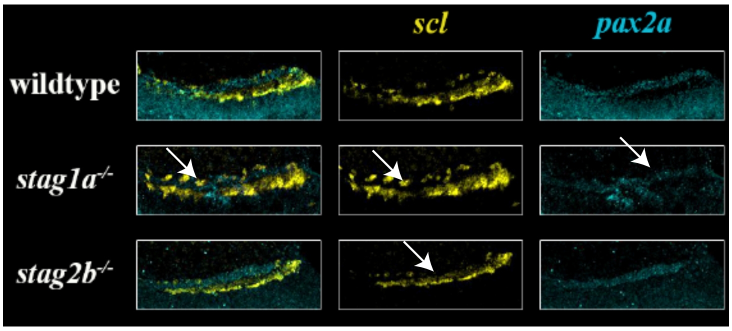

C

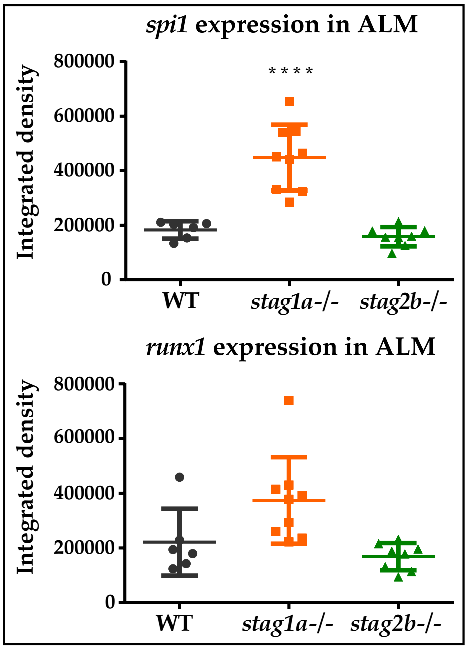

Supplementary Figure 5. Stag mutations affect gene expression in the posterior lateral mesoderm (PLM) and anterior lateral mesoderm (ALM). (A) Multiplexed *in situ* HCR of *scl* (yellow), *gata1* (red), and *fli1* (blue) expression at 15 hpf. Dorsal PLM views are shown, anterior to the top. Extra *scl* expression in *stag1a-/-* embryos and reduction of PLM expression in *stag2b-/-* embryos is marked by white arrows. Quantitative analysis of fluorescence integrated densities indicates *scl*, *gata1* and *fli1* trend to non-significant upregulation in *stag1a-/-* embryos in the PLM. Composition of the PLM is equivalent in all embryos. **(B)** Multiplexed *in situ* HCR of *scl* (yellow) and *pax2a* (cyan) expression at 15 hpf. Posterior views of a single PLM stripe are shown, dorsal to the left. Arrows mark lateral expansion of *scl* into the *pax2a* domain in the middle *stag1a-/-* panels and reduced *scl* expression in the lower *stag2b-/-* panel. **(C)** Quantitative analysis of fluorescence integrated densities of ALM markers shows an increase of *runx1* and *spi1* expression in *stag1a-/-* embryos. **** $P \leq 0.0001$; one-way ANOVA.
