# Supplementary material for: Cohesin Components Stag1 and Stag2 Differentially Influence Haematopoietic Mesoderm Development in Zebrafish Embryos
Source: Front Cell Dev Biol. 2020 Dec 7;8:617545. doi: 10.3389/fcell.2020.617545 (PMC7750468; doi:10.3389/fcell.2020.617545)
Supplement: Supplementary file 6 [file Data_Sheet_6.PDF]

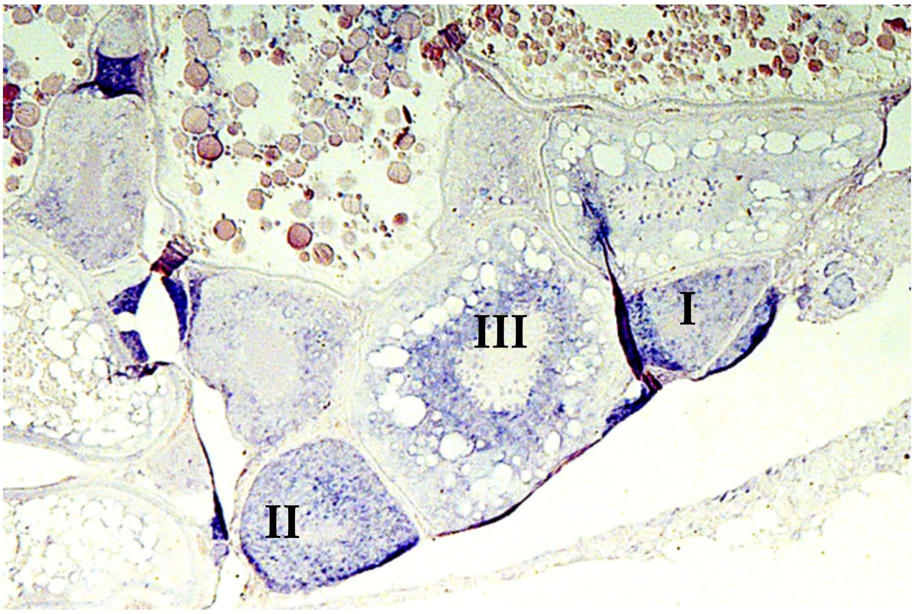

Supplementary Figure 6. *in situ* hybridization of *stag2a* in adult zebrafish ovary. Expression of *stag2a* as detected by *in situ* hybridization (blue/purple) is clearly visible in Stage I, II and III oocytes in this transverse section through wild type adult zebrafish ovary.
