# Supplementary material for: Cohesin Components Stag1 and Stag2 Differentially Influence Haematopoietic Mesoderm Development in Zebrafish Embryos
Source: Front Cell Dev Biol. 2020 Dec 7;8:617545. doi: 10.3389/fcell.2020.617545 (PMC7750468; doi:10.3389/fcell.2020.617545)
Supplement: Supplementary file 7 [file Data_Sheet_7.PDF]

Supplementary Tables

Supplementary Table 1. List of accession identifiers for proteins used for phylogenetic analysis.

Protein	Accession ID
Hs STAG1	NP_005853.2
Hs STAG2	NP_001036214.1
Gg STAG1	XP_015146838.1
Gg STAG2	XP_004940885.1
Mm Stag1	NP_001344193.1
Mm Stag2	NP_001071180.1
Xt stag1	NP_001121432.1
Xt stag2	XP_002931833.2
Dr stag1a	NP_001349269.1
Dr stag1b	XP_692120.3
Dr stag2a	NP_001093498.1
Dr stag2b	XP_005173250.1

Supplementary Table 2. sgRNA sequences used to generate CRISPR mutants. PAM sequences are marked in blue.

Target gene	Sequence 5'-3'	CHOPCHOP *in silico* efficiency
stag1a	GGGCTTTATGGCAGTCCAGAGGG	49.5
stag1b	CGGGAGGAGGCCGAATGGAGTGG	54.11
stag2b	GGCCCTGGAGAGAAGGGAAAAGG	45.19

Supplementary Table 3. Primer sequences used in this study.

Target gene	Forward primer	Reverse primer
	**Primers used for qPCR**	
stag1a	CTGGACCTTACATGACCGGC	TATCCAGCGTCATGGACACG
stag1b	CCAGGTTGATGCAGAAAAGGTG	GGCGTCCAGATGCTTTTCCAT
stag2a	AGCCGCTTCAAGGATCGAAT	CAGCGTCAGCAGCTTAATGG
stag2b	CAATAGCAGAGATCCGGGCG	GACACTTCAGACGCACCTCA
gata1a	TTACTGCCACCCGTTGATGT	TTGGCGAACTGGACTGTGTC
	**Primers used for *in situ* hybridisation**	
stag1a	CTTTGCCCTCACCTTCGGAT	GAGTTCTGCTCTCTCTCGCC
stag1b	GTCTGAAGCATTCTGGGGCT	GGCATCCCTGTAACGGTGAA
stag2a	AAGGGCGAAATGGCAAACTT	GACGCACCTCACCTTGCTTA
stag2b	CATCCTCACTGTTGGCCTGT	GACACTTCAGACGCACCTCA
	**Primers used for genotyping**	
stag1a CRISPR	GCCTCGGAAGTCTCCATCAG	GCACACCTGCATAGCACTCT
stag1b CRISPR	GGCGGCTAATAAGAAGGCCA	AAGCAGCACACAACCTCGAA
stag2b CRISPR	GTCACTCTGCTTCAGGCGAA	TGACCTGCATGGCACTCTTC

Supplementary Table 4. Morpholino sequences used in this study.

Name	Sequence 5'-3'	Binding site
stag1a	GGTTAGATGTTGTGTTACAGGTCT	5'UTR
stag2b	GTAATTCCGGTGCGGCTATCATTTC	ATG
